# Lung Ultrasound Abnormalities and LUS Score After COVID-19 Pneumonia: Determinants and Associations with Dyspnoea in a Prospective Cohort

**DOI:** 10.3390/jcm15093438

**Published:** 2026-04-30

**Authors:** Francisco Navarro-Romero, Cristina Asencio-Méndez, Francisco Rivas-Ruiz, Blanca Sánchez-Mesa, María Dolores Martín-Escalante, Julián Olalla-Sierra

**Affiliations:** 1 Department of Internal Medicine, Costa del Sol University Hospital, 29630 Marbella, Spain; 2 Faculty of Medicine, University of Malaga, 29071 Malaga, Spain; 3 Research and Innovation Unit, Costa del Sol University Hospital, 29630 Marbella, Spain; 4 IBIMA Platform BIONAND, Instituto de Investigación Biomédica de Málaga (IBIMA), 29590 Malaga, Spain; 5 Red de Investigación en Cronicidad, Atención Primaria y Promoción de la Salud (RICAPPS), 28029 Madrid, Spain

**Keywords:** COVID-19, severe pneumonia, diagnostic technology, lung ultrasound, LUS score, dyspnoea, post-COVID-19, pulmonary sequelae

## Abstract

**Background/Objectives**: The clinical determinants and functional relevance of persistent lung ultrasound (LUS) abnormalities after COVID-19 pneumonia remain poorly characterized. We aimed to identify determinants of qualitative LUS abnormalities and global lung involvement assessed by the LUS score, and to evaluate their association with persistent dyspnoea. **Methods**: We conducted a prospective observational study that included 261 patients who were hospitalized for COVID-19 pneumonia and were assessed 1–6 months after discharge. A standardized 14-zone LUS protocol was used to assess qualitative abnormalities (pleural line irregularity, ≥3 B-lines, and subpleural consolidations) and to calculate the LUS score. Associations with clinical variables, including dyspnoea assessed by the modified Medical Research Council (mMRC) scale, were analyzed using multivariable logistic regression. **Results**: The severity of the acute pneumonia episode emerged as the strongest determinant of qualitative LUS abnormalities and elevated LUS score (>6). Increasing age was independently associated with ultrasound findings. Persistent dyspnoea (mMRC ≥ 1) was associated with all qualitative abnormalities and with a higher prevalence of elevated LUS score (56.6% vs. 22.1%; *p* < 0.001). A graded association was observed between dyspnoea severity and both qualitative findings and LUS score. An increase in dyspnoea from baseline (ΔmMRC ≥ 1) remained independently associated with an elevated LUS score. **Conclusions**: Persistent LUS abnormalities are strongly associated with the severity of the acute episode. The LUS score provides a robust, clinically meaningful measure of residual lung involvement and shows a stronger association with persistent dyspnoea than qualitative findings, supporting its role in follow-up and risk stratification.

## 1. Introduction

Severe acute respiratory syndrome coronavirus 2 (SARS-CoV-2) infection is frequently complicated by pneumonia, which may be severe in a substantial proportion of patients and may progress to acute respiratory distress syndrome, with potentially life-threatening consequences [1]. Since the onset of the pandemic, COVID-19 has affected hundreds of millions of individuals worldwide and has caused more than 6.6 million deaths [2,3].

Beyond the acute phase, a considerable proportion of patients develop persistent symptoms and, in some cases, residual structural lung abnormalities. A systematic review and meta-analysis reported persistent fatigue in 28% of patients and dyspnoea in 9.4% two years after infection [4]. Imaging studies have demonstrated residual pulmonary involvement months after SARS-CoV-2 infection, predominantly characterized by ground-glass opacities, consolidations, and reticulations on chest computed tomography (CT) [5].

The high number of affected individuals and the prevalence of persistent respiratory symptoms highlight the need for accessible tools for post-COVID-19 follow-up. In this context, lung ultrasound offers several advantages, including wide availability, bedside applicability, low cost, and the absence of ionizing radiation [6,7,8]. In patients with interstitial lung involvement, it typically shows pleural line irregularities and vertical artifacts (B-lines), which correlate well with CT findings [9,10,11].

Several studies have reported persistent lung ultrasound abnormalities following COVID-19 [12,13,14]. Systematic reviews have identified pleural line irregularity, B-lines, and subpleural consolidations as the most frequent findings, with higher prevalence in patients with more severe disease, a tendency to decrease over time, and good concordance with CT findings [15,16]. However, these studies are limited by heterogeneity in ultrasound protocols, timing of follow-up, and sample size, and—most importantly—the clinical determinants of persistent ultrasound abnormalities remain insufficiently characterized.

In a previous study from our cohort, we described the prevalence and distribution of residual lung ultrasound abnormalities during the first six months after hospitalization for COVID-19 [17]. In the present study, we aimed to identify the clinical determinants of persistent qualitative lung ultrasound abnormalities (pleural line irregularity, B-lines, and subpleural consolidations) and of the global burden of lung involvement assessed by the LUS score, and to evaluate their association with persistent dyspnoea at follow-up.

## 2. Materials and Methods

### 2.1. Study Design and Population

We conducted a prospective observational study that included patients who were hospitalized for COVID-19 pneumonia at Hospital Universitario Costa del Sol (Marbella, Spain) between March 2020 and May 2022. Eligible patients were those who survived the index episode and attended a post-COVID-19 follow-up visit between 1 and 6 months after hospital discharge.

Inclusion criteria were: age ≥ 18 years; hospital admission for clinically and radiologically confirmed COVID-19 pneumonia, based on chest imaging (chest X-ray and/or computed tomography) showing findings consistent with SARS-CoV-2 infection; microbiological confirmation by RT-PCR and/or rapid antigen testing for SARS-CoV-2; follow-up assessment between 1 and 6 months after discharge; and availability of a complete lung ultrasound examination using a standardized 14-zone protocol.

The characteristics of the cohort and follow-up procedures have been previously described [17]. In the present study, an integrated evaluation of persistent lung ultrasound abnormalities was performed, including both qualitative assessment and quantification of lung involvement using the LUS score.

This study was designed and reported in accordance with the Strengthening the Reporting of Observational Studies in Epidemiology (STROBE) recommendations [18]. Ethical approval and the requirement for written informed consent were waived by the Ethics Committee of Hospital Universitario Costa del Sol, in accordance with national regulations applicable to observational studies using anonymized data.

### 2.2. Lung Ultrasound Assessment

Lung ultrasound examinations were performed using a SonoSite LX system (FUJIFILM SonoSite, Bothell, WA, USA) with a linear (≥7 MHz) and a convex (2.5–5 MHz) probe, following a standardized 14-zone protocol according to Soldati et al. [19].

All examinations were performed by two experienced investigators, with systematic image acquisition and independent review by a second observer. In case of disagreement, a consensus was reached. Although formal interobserver agreement analysis was not performed, this procedure was implemented to enhance the consistency and reliability of image interpretation.

In each lung region, the following findings were recorded: normal pleural line with A-lines, pleural line irregularity, ≥3 B-lines, and subpleural consolidations (<1 cm or ≥1 cm). Each finding was analyzed independently.

A quantitative evaluation of lung involvement was performed using the LUS score, calculated across the 14 explored lung regions according to the system proposed by Soldati et al. [19]. Each region was scored from 0 to 3: 0 (normal pleural line with A-lines), 1 (irregular pleural line with B-lines), 2 (pleural discontinuity with multiple B-lines and small subpleural consolidations), and 3 (extensive hyperechoic areas with or without larger consolidations).

The sum of all regional scores yielded a total LUS score (range 0–42), used as a measure of residual lung involvement. For categorical analyses, an elevated LUS score was defined as >6 in the absence of validated cut-offs in the post-COVID-19 setting. This threshold was derived from the cohort distribution and corresponded to the 25th percentile among patients with more severe pneumonia (moderate to very severe), with the aim of conservatively identifying patients with a higher burden of residual lung involvement.

### 2.3. Clinical and Laboratory Variables

Demographic, anthropometric, and clinical variables were collected. Overweight and obesity were defined according to World Health Organization (WHO) criteria [20].

Acute episode variables included length of hospital stay, admission to the intensive care unit (ICU), and pneumonia severity. Severity was assessed using the MuLBSTA prognostic score (multilobar infiltrates, lymphopenia, bacterial co-infection, smoking history, hypertension, and age ≥ 65 years) [21], and according to the maximum level of oxygen support required during the acute phase. Oxygen support was categorized as very mild (no oxygen therapy), mild with nasal cannula at 1–3 L/min, moderate with nasal cannula at 4–7 L/min or face mask with fraction of inspired oxygen (FiO_2_) 0.32–0.60, severe with non-rebreather mask ≥8 L/min or high-flow nasal oxygen (HFNO), and very severe with non-invasive mechanical ventilation (NIMV) or invasive mechanical ventilation (IMV).

Dyspnoea was assessed using the modified Medical Research Council (mMRC) scale (0 = no dyspnoea, 4 = severe dyspnoea) [22], recording both baseline (pre-infection) and follow-up values; change was defined as the difference between the two measurements. Other persistent symptoms and laboratory parameters from the first 72 h of admission, including lymphocyte count, D-dimer, CRP, lactate dehydrogenase (LDH), ferritin, and interleukin-6 (IL-6) were also collected.

### 2.4. Statistical Analysis

A descriptive analysis of the cohort was performed. Continuous variables are presented as mean ± standard deviation (SD) or median and interquartile range (IQR), depending on data distribution, and categorical variables as frequencies and percentages.

Univariable associations were assessed using Student’s t-test or the Mann–Whitney U test for continuous variables, and the χ^2^ test or Fisher’s exact test for categorical variables, as appropriate. To identify factors associated with each qualitative ultrasound finding (pleural line irregularity, ≥3 B-lines, and subpleural consolidations) and with an elevated LUS score (>6), independent multivariable logistic regression models were constructed.

Variables with *p* < 0.05 in univariable analysis and those considered clinically relevant a priori (age, sex, severity of the acute episode, and respiratory comorbidities) were included in the models. Results are expressed as odds ratios (ORs) with 95% confidence intervals (95% CI). Model calibration was assessed using the Hosmer–Lemeshow test.

A two-sided p-value < 0.05 was considered statistically significant. Analyses were performed using IBM SPSS Statistics version 29.0.2.0 (IBM Corp., Armonk, NY, USA).

## 3. Results

### 3.1. Study Population Characteristics

**Figure 1:**
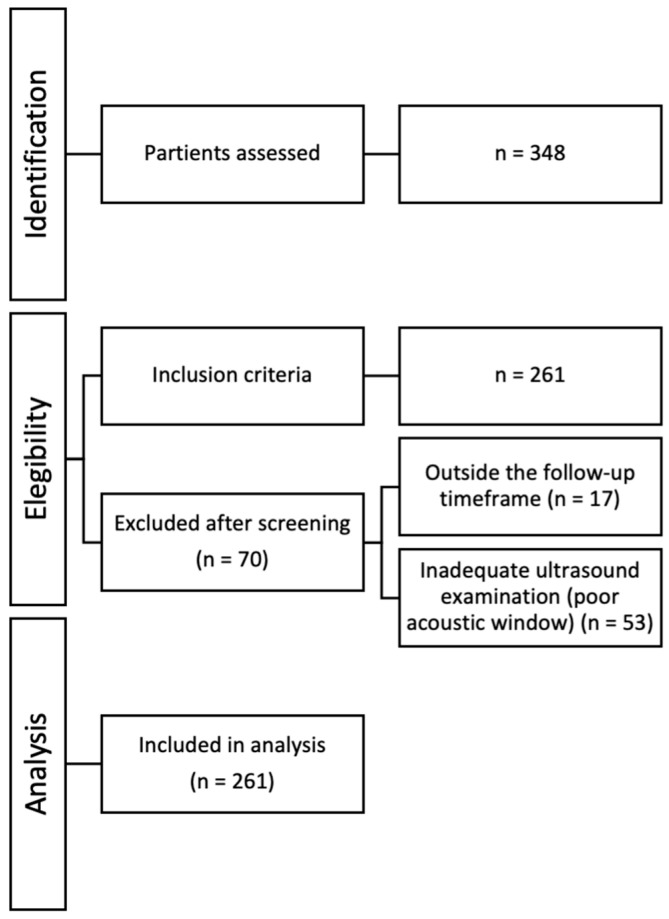
Flowchart of patient selection and follow-up (1–6 months after hospital discharge).

During the study period, 348 patients attended the post-COVID-19 follow-up clinic, of whom 261 met the inclusion criteria and were included in the analysis (Figure 1). Excluded patients did not differ in age, sex, or severity of the acute pneumonia episode.

The median time from hospital discharge to evaluation was 75 days (IQR: 62–88), corresponding to mid-term follow-up. The temporal distribution of evaluations was as follows: 63 patients (24.1%) were assessed between 30 and 60 days, 173 (66.3%) between 61 and 120 days, and 24 (9.2%) between 121 and 180 days after discharge. The mean age was 64.9 ± 14.0 years, and 54.8% were male.

Regarding the severity of the acute episode, 18.8% did not require oxygen therapy, 37.9% received low-flow nasal cannula, 23.0% received oxygen via face mask, 13.0% required a non-rebreather mask or high-flow nasal oxygen, and 7.3% required IMV and ICU admission.

The mean MuLBSTA score was 9.4 ± 4.0, with 31.0% scoring ≥ 12. At follow-up, 67.0% of patients reported dyspnoea (mMRC ≥ 1), and 45.5% showed worsening compared with baseline.

Baseline characteristics have been previously reported [17]; Table 1 summarizes the variables most relevant to the present analysis.

### 3.2. Overview of Lung Ultrasound Findings

To improve interpretability, results are presented according to three main domains: qualitative lung ultrasound findings, quantitative assessment using the LUS score, and their relationship with clinical outcomes.

At follow-up, a high proportion of patients showed persistent lung ultrasound abnormalities, with pleural line irregularity being the most frequent finding, followed by B-lines and subpleural consolidations. These descriptive findings have been previously reported [17].

Quantitative assessment showed a mean LUS score of 6.3 ± 5.3, with a median of 6 (IQR: 2–10). A total of 54 patients (20.7%) had a LUS score of 0, while the maximum value observed was 21 (1.1%). Overall, 34.1% of patients had scores between 1 and 6, another 34.1% between 7 and 13, and 11.0% between 14 and 21, reflecting substantial variability in the burden of residual lung involvement.

### 3.3. Determinants of Qualitative Lung Ultrasound Abnormalities

Demographic factors were significantly associated with persistent ultrasound abnormalities. Patients with abnormal findings were older across all qualitative patterns. Mean age was 66.6 vs. 59.4 years for pleural line irregularity (*p* < 0.001), 69.5 vs. 62.0 for ≥3 B-lines (*p* < 0.001), and 67.1 vs. 63.7 for subpleural consolidations (*p* < 0.05). Male sex was associated with pleural line irregularity (83.9% vs. 66.1%; *p* = 0.04) and ≥3 B-lines (51.0% vs. 22.9%; *p* < 0.001).

Body composition showed a modest association, with lower body mass index observed in patients with pleural line irregularity and subpleural consolidations, while no significant difference was observed for ≥3 B-lines.

Among comorbidities, chronic obstructive pulmonary disease (COPD) showed the strongest association with ≥3 B-lines (91.7% vs. 35.7%; *p* < 0.001) and subpleural consolidations (66.7% vs. 34.1%; *p* = 0.047), whereas arterial hypertension was associated with pleural irregularity and ≥3 B-lines.

**Figure 2:**
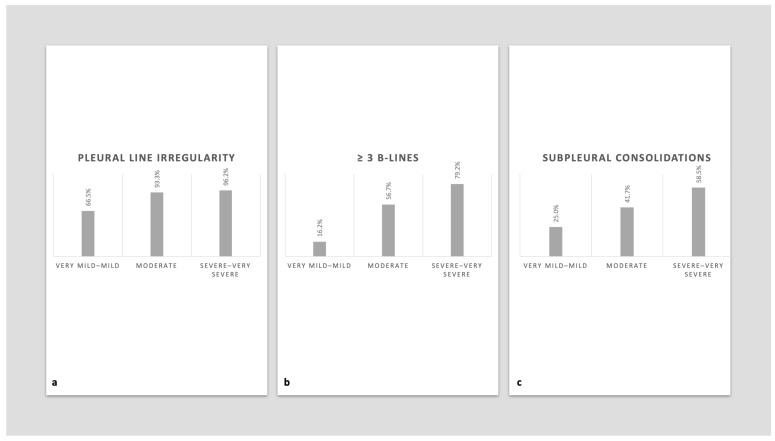
Distribution of lung ultrasound findings according to the severity of the acute COVID-19 pneumonia episode. (**a**) Proportion of patients with pleural line irregularity according to pneumonia severity. (**b**) Proportion of patients with ≥3 B-lines per lung region according to pneumonia severity. (**c**) Proportion of patients with subpleural consolidations according to pneumonia severity. A progressive increase in the frequency of all ultrasound abnormalities was observed with increasing severity of the acute episode (*p* for trend < 0.001 for all comparisons).

Markers of acute disease severity were consistently associated with all qualitative ultrasound findings. Using the three-category severity classification (very mild–mild, moderate, and severe–very severe), a clear progressive increase in abnormalities was observed across severity levels (Figure 2).

Similarly, higher MuLBSTA scores were observed in patients with persistent abnormalities, both in continuous and categorical analyses (MuLBSTA ≥ 12; *p* < 0.001).

Biological parameters also followed this pattern, with a higher prevalence of lymphopenia and increased levels of D-dimer, LDH, and ferritin among patients with abnormal ultrasound findings.

Factors associated with each ultrasound variable are summarized in Table 2, with the full analysis presented in Supplementary Table S1.

### 3.4. Determinants of Elevated LUS Score

**Figure 3:**
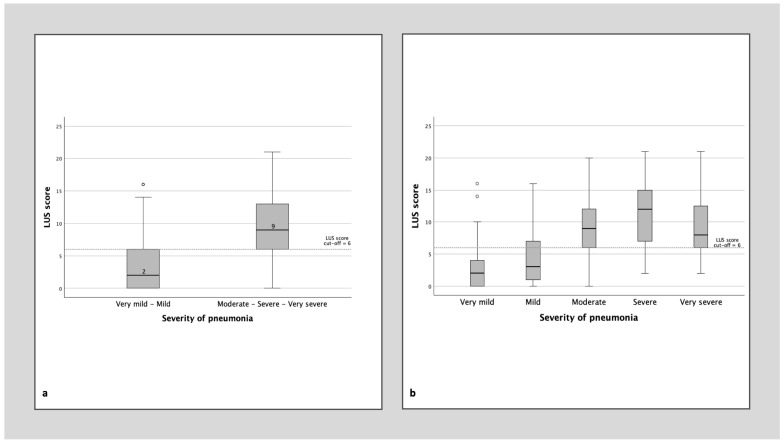
Association between pneumonia severity during the acute episode and lung ultrasound findings at follow-up. (**a**) Distribution of the LUS score according to pneumonia severity, grouped into very mild–mild and moderate–severe–very severe categories. (**b**) Distribution of the LUS score across the five predefined categories of pneumonia severity (very mild, mild, moderate, severe, and very severe). The boxplots represent the median and interquartile range (IQR), with whiskers indicating the range of observed values. The dashed horizontal line indicates the cut-off used to define an elevated LUS score (>6).

The LUS score showed a strong and consistent association with disease severity. Patients with more severe pneumonia had significantly higher LUS scores (median 9 vs. 2; *p* < 0.001). This relationship is illustrated in Figure 3, which shows a progressive increase in LUS score across severity categories.

Demographic characteristics followed a similar pattern. Patients with a LUS score > 6 were older (69.7 vs. 60.9 years; *p* < 0.001) and more frequently male (*p* < 0.001). A modest but statistically significant difference in body mass index was also observed, with lower values in patients with elevated scores (*p* = 0.011).

Comorbidities associated with elevated LUS score included arterial hypertension, ischemic heart disease, and COPD.

**Figure 4:**
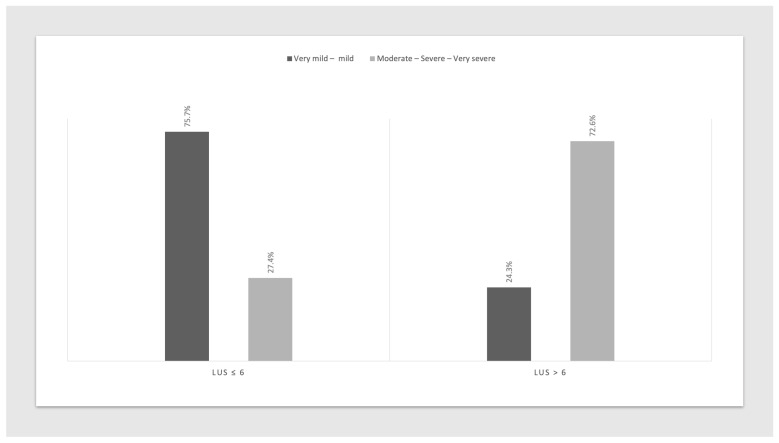
Relationship between LUS score categories at follow-up and the severity of the initial pneumonia episode. Distribution of patients with LUS score ≤ 6 and LUS score > 6 according to grouped pneumonia severity categories (very mild–mild vs. moderate–severe–very severe). A higher proportion of patients with LUS score > 6 was observed among those with more severe pneumonia, supporting a clear severity-dependent pattern.

In line with the qualitative findings, acute disease severity showed a clear graded association with LUS score (*p* < 0.001; Figure 4). Patients with elevated LUS scores also had longer hospital stays, longer duration of oxygen therapy, and higher MuLBSTA scores (*p* < 0.001).

From an analytical perspective, lymphopenia and higher levels of D-dimer, LDH, ferritin, and IL-6 were observed (*p* < 0.05).

Results are summarized in Table 3, with the complete analysis provided in Supplementary Table S2.

### 3.5. Association Between Lung Ultrasound Abnormalities and Persistent Dyspnoea

Persistent dyspnoea at follow-up (mMRC ≥ 1) was associated with both qualitative and quantitative ultrasound findings. Patients with dyspnoea had a higher frequency of pleural line irregularity (80.6% vs. 66.3%; *p* = 0.017), ≥3 B-lines (44.6% vs. 25.6%; *p* = 0.005), and subpleural consolidations (41.7% vs. 23.3%; *p* = 0.005).

Similarly, patients with dyspnoea were more likely to have an elevated LUS score (>6) (56.6% vs. 22.1%; *p* < 0.001). In addition, worsening dyspnoea compared to baseline was consistently associated with a progressive increase in ultrasound abnormalities. For instance, the frequency of ≥3 B-lines increased from 33.1% in patients without worsening dyspnoea (ΔmMRC = 0) to 60.7% in those with moderate worsening (ΔmMRC = 2), while the proportion of patients with LUS score > 6 increased from 33.1% to 71.4% across the same categories.

Overall, both qualitative and quantitative ultrasound abnormalities increased progressively across higher levels of dyspnoea severity (see Supplementary Figure S1).

These univariable findings were partially confirmed in the multivariable analysis, as shown below.

### 3.6. Independent Determinants of Persistent Lung Ultrasound Abnormalities

In multivariable analysis, the severity of the acute episode emerged as the main determinant of persistent lung ultrasound abnormalities.

Pleural line irregularity was independently associated with age (OR 1.038; 95% CI 1.012–1.064; *p* = 0.004) and pneumonia severity (OR 10.46; 95% CI 4.19–26.12; *p* < 0.001).

For ≥3 B-lines per lung region, independent determinants were age, male sex, COPD, and pneumonia severity.

For subpleural consolidations, pneumonia severity was the main determinant, with an additional association with weight loss during follow-up.

For an elevated LUS score (>6), independent determinants were pneumonia severity (OR 10.15; 95% CI 4.80–21.43; *p* < 0.001) and age (OR 1.07; 95% CI 1.03–1.10; *p* < 0.001). In addition, an increase in dyspnoea from baseline (ΔmMRC ≥ 1) was independently associated with an elevated LUS score (OR 5.62; 95% CI 2.58–12.22; *p* < 0.001).

Full multivariable results are shown in Table 4.

## 4. Discussion

In this prospective observational study, we systematically evaluated residual lung involvement during the first six months after COVID-19 pneumonia using an integrated ultrasound approach, combining qualitative assessment of key findings—pleural line irregularity, B-lines, and subpleural consolidations—with global quantification through the LUS score. Our results provide insight into the clinical determinants of these abnormalities and their clinical relevance, including their association with persistent dyspnoea during follow-up.

The severity of the acute episode consistently emerged as the main determinant of persistent lung ultrasound abnormalities, both in qualitative findings and in the LUS score, remaining independently associated across all multivariable models. This effect was particularly pronounced for pleural line irregularity, the presence of ≥3 B-lines, and LUS score > 6, whereas it was less marked for subpleural consolidations, suggesting a differential sensitivity of these findings to the severity of acute lung injury and their potential value as markers of residual disease burden.

These findings further reinforce the link between the severity of acute lung injury and the persistence of structural abnormalities detected by lung ultrasound and support the role of the LUS score as a robust quantitative marker of residual alveolar–interstitial involvement. In this regard, our results are consistent with previous studies reporting a higher burden of B-lines in patients with more severe acute respiratory disease [23,24], particularly among those who required non-invasive ventilation or intensive care unit admission [25,26]. Similarly, other studies have reported higher LUS scores in patients with longer hospital stays or in those who required ventilatory support [27,28], as well as in patients with more severe pneumonia during the acute phase [14,29].

Beyond acute disease severity, host-related factors also played a relevant role. Age was independently associated with multiple ultrasound findings and with the LUS score, supporting its role as the main baseline determinant of residual lung involvement. In contrast to previous studies, where this association was mainly observed in univariable analyses [28,30], our findings confirm its independence after multivariable adjustment, suggesting a role as a marker of reduced pulmonary recovery capacity.

Male sex was associated with pleural line irregularity and ≥3 B-lines in univariable analysis, in line with previous reports [14], but not with subpleural consolidations. However, after multivariable adjustment, this association persisted only for ≥3 B-lines, suggesting that the effect of male sex on other ultrasound abnormalities may be partly mediated by differences in acute disease severity or other clinical factors.

In line with this, emerging evidence suggests that patient-related biological heterogeneity, including sex-related differences and baseline vulnerability such as frailty, may significantly influence COVID-19 outcomes and recovery trajectories. Females have been shown to exhibit lower radiological lung involvement, attenuated inflammatory responses, and improved clinical outcomes despite a higher prevalence of frailty, supporting the role of intrinsic biological factors beyond traditional risk markers [31]. Moreover, frailty reflects a multidimensional state of reduced physiological reserve that may modulate both disease expression and recovery patterns [32]. These observations highlight that individual susceptibility—not fully captured by acute severity or comorbidity burden alone—may contribute to variability in lung ultrasound findings and their clinical implications.

Comorbidities showed specific associations depending on the type of ultrasound finding, with COPD being the main factor associated with ≥3 B-lines. However, these findings should be interpreted with caution due to the limited number of patients with COPD, and they did not remain independently associated with the LUS score after adjustment, suggesting that their effect may be mediated by the severity of the acute episode. Notably, previous studies in the post-COVID-19 setting have not demonstrated a significant association between COPD and LUS abnormalities, although these analyses were similarly limited by small sample sizes. Therefore, LUS abnormalities in this population should be interpreted within the appropriate clinical context.

Similarly, inflammatory and thrombotic biomarkers (lymphopenia, D-dimer, LDH, ferritin, and IL-6) were associated with ultrasound abnormalities in univariable analyses but did not remain independent determinants, supporting their role as markers of acute severity rather than of residual lung damage per se. These associations may be related to thrombotic, inflammatory, and microvascular injury processes during the acute phase, as well as to incomplete resolution of interstitial involvement in later stages.

From a clinical perspective, one of the most relevant findings is the consistent association between lung ultrasound abnormalities and persistent dyspnoea, with a progressive increase in ultrasound abnormalities across increasing levels of symptom severity.

Of particular clinical relevance, in multivariable models, persistent dyspnoea was independently associated only with an elevated LUS score (>6), suggesting that integrated quantification of lung involvement more robustly captures functional impairment than the isolated assessment of qualitative findings. These results are consistent with previous studies [13,27], but extend current evidence by demonstrating a graded association with both mMRC grade and change from baseline, further supporting the role of the LUS score as an integrative marker of structural damage and symptom burden.

However, these findings should be interpreted with caution. Dyspnoea in the post-COVID-19 setting is a complex and multifactorial symptom that is often only partially explained by structural lung abnormalities. Factors such as physical deconditioning, cardiovascular involvement, frailty, and psychological components may contribute substantially. In this context, some degree of discordance between imaging findings and clinical symptoms should be expected, and lung ultrasound abnormalities are likely to explain only part of the observed symptom burden. Although the assessment of both dyspnoea at follow-up and changes relative to baseline (ΔmMRC) may have helped to mitigate the influence of pre-existing conditions and other confounding factors, the observed relationships should be interpreted as associations rather than implying causality, particularly in the context of the limited representation of the most severe cases in our cohort.

Within this framework, it is relevant to consider whether lung ultrasound provides prognostic information beyond that conveyed by the severity of the acute episode. While our findings support a strong association between LUS abnormalities and initial disease severity, the extent to which LUS adds independent prognostic value for long-term pulmonary outcomes remains to be fully established. This distinction is particularly important when interpreting LUS as either a marker of residual structural damage or as a surrogate of prior disease severity.

From a pathophysiological perspective, although lung ultrasound abnormalities likely reflect some degree of residual pulmonary involvement, their exact correlate in the post-acute phase remains uncertain. In patients with mild or persistent symptoms, these findings may represent a combination of residual, non-specific, or subclinical alterations and ongoing recovery processes rather than established structural lung damage. By contrast, in patients with a higher symptom burden or a history of more severe acute disease, the persistence of ultrasound abnormalities may be more indicative of clinically relevant residual lung involvement.

In this context, further studies integrating advanced imaging techniques, pulmonary function testing, and patient-reported outcomes such as quality of life will be important to better characterize the clinical significance of these findings. In addition, validation of these results in other clinical settings will be essential to strengthen the external validity and generalizability of this approach.

Among the study’s limitations, lung ultrasound is an operator-dependent technique. To improve consistency and reproducibility, examinations were performed using a standardized protocol, including systematic image acquisition, storage of key images, and independent review by a second observer. When discrepancies were identified, images were re-evaluated to ensure consistency in interpretation.

Although this approach was implemented to ensure consistency and accuracy in image interpretation, no formal interobserver agreement analysis (e.g., kappa index) was performed. This may limit reproducibility; however, the standardized protocol and image review process likely helped to mitigate this effect. Formal assessment of interobserver agreement should be considered in future studies.

In addition, systematic chest CT was not performed. Lung ultrasound was conducted as part of the routine follow-up visit, independently of whether additional imaging studies such as chest radiography or CT were requested based on clinical judgment. Therefore, although some patients may have undergone complementary imaging, no standardized or systematic comparison with reference imaging techniques was available. This limits the ability to assess the concordance between ultrasound findings and other radiological patterns within our cohort.

Nevertheless, available evidence supports the validity of lung ultrasound in this context. In this regard, Giovanetti et al. reported a high diagnostic performance of lung ultrasound compared with chest CT for the identification of interstitial patterns in post-COVID-19 follow-up, with an area under the curve (AUC) of 0.94 and good agreement between techniques (Cohen’s kappa = 0.74) [12]. Similarly, Russo et al. demonstrated an association between LUS score and fibrotic-like changes on chest CT at six months after the acute episode [30]. Consistent findings have also been reported in studies with shorter follow-up periods of 2–5 months [33,34], as well as in studies including longer-term assessments at six months and one year [35].

Furthermore, in patients with obesity, technical limitations inherent to thoracic ultrasound—such as increased chest wall thickness and ultrasound attenuation [36]—may have contributed to a lower detection of certain findings; however, examinations with inadequate acoustic windows were excluded, which likely mitigated, although did not completely eliminate, this potential bias.

The absence of a validated cut-off for the LUS score represents another limitation. The threshold used (>6) was derived from the cohort and corresponds to the 25th percentile of patients with more severe pneumonia (moderate to very severe), with the aim of conservatively identifying those with a higher burden of residual lung involvement. Moreover, it showed an adequate ability to discriminate clinically relevant outcomes, particularly persistent dyspnoea. However, variability in ultrasound protocols, scoring systems, and cut-off values across studies [27,28] limits direct comparability. Therefore, this threshold should be considered exploratory and interpreted within the context of the 14-zone protocol and the distribution of our cohort and requires external validation.

Finally, the cross-sectional design precludes assessment of the temporal evolution of lung ultrasound abnormalities and limits the ability to establish temporal or causal relationships between clinical variables and ultrasound findings. In addition, the observational nature of the study does not allow full adjustment for intercurrent clinical conditions arising during recovery, which may have influenced both symptoms and ultrasound findings at follow-up. Therefore, residual confounding cannot be excluded.

Despite these limitations, our study provides relevant evidence on the role of lung ultrasound in post-COVID-19 follow-up. Ultrasound evaluation identified persistent lung abnormalities with a considerable frequency at mid-term follow-up and showed a close relationship between the severity of the initial episode and the burden of residual lung involvement.

In this context, the LUS score emerges as a quantitative marker capable of integrating overall lung damage and reflecting its clinical impact, showing a stronger association with persistent dyspnoea than isolated qualitative findings. These findings suggest that ultrasound-based quantification may contribute to clinical risk stratification and to the identification of patients who could benefit from closer clinical or radiological follow-up. The accessibility, absence of radiation, and low cost of lung ultrasound [37], together with its good correlation with chest CT, support its role as an initial assessment tool in patients with persistent symptoms after COVID-19 pneumonia.

## 5. Conclusions

In conclusion, persistent lung ultrasound abnormalities at mid-term follow-up after COVID-19 pneumonia are closely associated with the severity of the initial episode. Both qualitative findings and quantitative assessment using the LUS score reflect the burden of residual lung involvement; however, an elevated LUS score (>6) emerges as the most clinically relevant parameter, as it is independently associated with persistent dyspnoea. These findings support the role of lung ultrasound as a practical, radiation-free tool for patient follow-up and suggest that LUS score-based quantification may help identify patients with clinically significant residual lung involvement.

## Figures and Tables

**Table 1:** Baseline demographic, laboratory, and acute pneumonia characteristics of the study population.

Variable	Result
**Demographic and anthropometric variables**	
Age, years	64.9 ± 14.0
Male sex, n (%)	143 (54.8)
**Acute pneumonia severity during hospitalization**	
Very mild (no oxygen therapy), n (%)	49 (18.8)
Mild (low-flow nasal cannula, 1–3 L/min), n (%)	99 (37.9)
Moderate (simple face mask, 4–7 L/min), n (%)	60 (23.0)
Severe (non-rebreather mask ≥ 8 L/min or HFNO), n (%)	34 (13.0)
Very severe (NIMV or IMV), n (%)	19 (7.3)
**Prognostic scores**	
MuLBSTA score	9.44 ± 4.02
MuLBSTA high-risk (12–22 points), n (%)	81 (31.0)
**Laboratory data during acute pneumonia**	
Leukocytes, cells/µL	7340 [4832–10,140]
Lymphocytes, cells/µL	815 [600–1202]
Neutrophils, cells/µL	5435 [3467–8480]
D-dimer, ng/mL	1077.5 [562–2633.7]
CRP, mg/dL	75.7 [34.2–145.5]
LDH, U/L	299 [240.5–380]
Ferritin, ng/mL	645.3 [302.4–1262.7]
IL-6, pg/mL	10.1 [4.3–23.1]

Abbreviations: HFNO, high-flow nasal oxygen; NIMV, non-invasive mechanical ventilation; IMV, invasive mechanical ventilation; CRP, C-reactive protein; LDH, lactate dehydrogenase; IL-6, interleukin-6.

**Table 2:** Factors associated with persistent lung ultrasound abnormalities (univariable analysis).

Variable		Pleural Line Irregularity			≥ 3 B-Lines per Region			Subpleural Consolidations		
		Present	Absent	*p*	Present	Absent	*p*	Present	Absent	*p*
**Baseline characteristics**										
Age, years		66.6 ± 13.1	59.4 ± 15.3	**<0.001**	69.5 ± 11.2	62.0 ± 14.8	**<0.001**	67.1 ± 13.1	63.7 ± 14.3	**<0.05**
Male sex, n (%)		120 (83.9)	23 (16.1)	**0.001**	73 (51.0)	70 (49.0)	**<0.001**	58 (40.6)	85 (59.4)	0.089
Female sex, n (%)		78 (66.1)	40 (33.9)	**0.001**	27 (22.9)	91 (77.1)	**<0.001**	35 (29.7)	83 (70.3)	0.089
Body mass index, kg/m^2^		28.1 ± 4.8	30.3 ± 6.7	**0.002**	28.1 ± 4.3	28.9 ± 5.9	0.120	27.7 ± 4.4	29.1 ± 5.8	**0.016**
Smoking history, n (%)		9 (47.4)	10 (52.6)	**0.006**	6 (31.6)	13 (68.4)	0.702	4 (21.1)	15 (78.9)	0.259
Arterial hypertension, n (%)		124 (82.1)	27 (17.9)	**0.009**	69 (45.7)	82 (54.3)	**0.006**	59 (39.1)	92 (60.9)	0.192
Chronic obstructive pulmonary disease, n (%)		12 (100)	0 (0)	0.076	11 (91.7)	1 (8.3)	**<0.001**	8 (66.7)	4 (33.3)	**0.047**
**Acute COVID-19 pneumonia characteristics**										
Severity of pneumonia n (%)	Very mild–mild	91 (61.5)	57 (38.5)	**<0.001**	24 (16.2)	124 (83.8)	**<0.001**	37 (25.0)	111 (75.0)	**<0.001**
	Moderate–very severe	107 (94.7)	6 (5.3)		76 (67.3)	37 (32.7)		56 (49.6)	57 (50.4)	
MuLBSTA score		10.2 ± 3.8	7.1 ± 3.6	**<0.001**	11.8 ± 3.2	7.9 ± 3.7	**<0.001**	10.6 ± 3.8	8.8 ± 3.9	**<0.001**
MuLBSTA ≥ 12		76 (93.8)	5 (6.2)	**<0.001**	58 (71.6)	23 (28.4)	**<0.001**	42 (51.9)	39 (48.1)	**<0.001**
**Laboratory parameters during acute phase**										
Lymphocytes, cells/mm^3^		770 (590–1135)	990 (670–1360)	**<0.001**	695 (532.5–920)	960 (650–1310)	**<0.001**	750 (590–1120)	850 (600–1270)	0.129
D-dimer, ng/mL		1256 (630–3759)	655 (429–1434)	**<0.001**	1810 (822–4115)	882 (502–1777)	**<0.001**	1811 (889–4967)	880 (506–1941)	**<0.001**
LDH, U/L		316 (247–411)	268 (202–340)	**<0.001**	337 (262–430)	281 (225–350)	**<0.001**	319.5 (249–402)	283 (236–371)	0.080

Data are presented as mean ± standard deviation, median [interquartile range], or number (%), as appropriate. Comparisons were performed using the χ^2^ test, Fisher’s exact test, or the Mann–Whitney U test, as appropriate. Statistically significant values (*p* < 0.05) are shown in bold. Abbreviations: LDH, lactate dehydrogenase.

**Table 3:** Factors associated with elevated LUS score (>6) (univariable analysis).

Variable		LUS Score ≤ 6	LUS Score > 6	*p*
**Baseline characteristics**				
Age, years		60.9 ± 14.8	69.7 ± 11.1	**<0.001**
Male sex, n (%)		63 (44.1)	80 (55.9)	**<0.001**
Female sex, n (%)		80 (67.8)	38 (32.2)	**<0.001**
Body mass index, kg/m2		29.3 ± 6.2	27.8 ± 4.1	**0.011**
Smoking history, n (%)		13 (68.4)	6 (31.6)	0.317
Arterial hypertension, n (%)		74 (49.0)	77 (51.0)	**0.038**
Ischaemic heart disease, n (%)		10 (34.5)	19 (65.5)	**<0.05**
Chronic obstructive pulmonary disease, n (%)		2 (16.7)	10 (83.3)	**<0.05**
**Acute COVID-19 pneumonia characteristics**				
Severity of pneumonia	Very mild–mild	112 (75.2)	36 (24.3)	**<0.001**
	Moderate–very severe	31 (27.4)	82 (72.6)	
MuLBSTA score, mean ± SD		7.67 ± 3.7	11.58 ± 3.2	**<0.001**
MuLBSTA score ≥ 12, n (%)		53 (29.4)	65 (80.2)	**<0.001**
Requirement for oxygen therapy, n (%)		98 (46.7)	112 (53.3)	**<0.001**
Duration of oxygen therapy, days, median (IQR)		5 (3–8.2)	8.5 (5–16)	**<0.001**
Length of hospital stay, days, median (IQR)		7 (5–11)	10.5 (6.75–16)	**<0.001**
**Laboratory parameters, median (IQR)**				
Lymphocytes (cells/mm^3^)		980 (670–1325)	690 (545–920)	**<0.001**
D-dimer (ng/mL)		790 (451–1676)	1830 (868–4202)	**<0.001**
LDH (U/L)		271 (219–350)	334.5 (266–429)	**<0.001**

Data are presented as mean ± standard deviation (SD), median (IQR), or number (percentage), as appropriate. Comparisons were performed using the χ^2^ test or Fisher’s exact test for categorical variables and Student’s t-test or Mann–Whitney U test for continuous variables, as appropriate. Statistically significant values (*p* < 0.05) are shown in bold. Abbreviations: SD, standard deviation; IQR, interquartile range; LDH, lactate dehydrogenase.

**Table 4:** Multivariable logistic regression analysis of factors associated with persistent lung ultrasound abnormalities and elevated LUS score at post-COVID-19 follow-up.

Variable	Pleural Line Irregularity OR (95% CI)	*p*	≥3 B-Lines per Lung Region OR (95% CI)	*p*	Subpleural Consolidations OR (95% CI)	*p*	LUS Score > 6 OR (95% CI)	*p*
Age (per year)	1.038 (1.012–1.064)	**0.004**	1.039 (1.013–1.066)	**0.003**	-	-	1.07 (1.03–1.10)	**<0.001**
Male sex	-	-	2.02 (1.02–4.00)	**0.043**	-	-	-	-
Moderate to very severe pneumonia ^1^	10.46 (4.19–26.12)	**<0.001**	8.02 (4.08–15.75)	**<0.001**	2.54 (1.44–4.46)	**0.001**	10.15 (4.80–21.43)	**<0.001**
Chronic obstructive pulmonary disease (COPD)	-	-	11.51 (1.30–101.33)	**0.028**	-	-	-	-
Weight loss during follow-up	-	-	-	-	3.04 (1.41–6.54)	**0.004**	-	-
ΔmMRC ≥ 1 during follow-up	-	-	-	-	-	-	5.62 (2.58–12.22)	**<0.001**

OR: odds ratio; CI: confidence interval. Models were adjusted for variables with *p* < 0.05 in univariable analysis and clinically relevant covariates (age, sex, acute disease severity, and respiratory comorbidities). ^1^ Moderate to very severe pneumonia compared with very mild to mild pneumonia. ΔmMRC ≥ 1 indicates worsening dyspnoea from baseline. Dashes indicate variables not retained in the final multivariable model. Statistically significant values (*p* < 0.05) are shown in bold.

## Data Availability

The datasets generated and/or analyzed during the current study are available from the corresponding author upon reasonable request.

## Supplementary Materials

The following supporting information can be downloaded at: https://www.mdpi.com/article/10.3390/jcm15093438/s1, Table S1: Clinical, laboratory, acute-phase, and symptom-related factors associated with persistent lung ultrasound abnormalities (univariable analysis); Table S2: Clinical, laboratory, acute-phase, and symptom-related factors associated with elevated LUS score (>6) (univariable analysis); Figure S1: Relationship between worsening dyspnoea and residual lung ultrasound findings at follow-up.

## Abbreviations

The following abbreviations are used in this manuscript:

LUS	lung ultrasound
COVID-19	coronavirus disease 2019
mMRC	modified Medical Research Council
SARS-CoV-2	severe acute respiratory syndrome coronavirus 2
CT	computed tomography
STROBE	Strengthening the Reporting of Observational Studies in Epidemiology
CRP	C-reactive protein
WHO	World Health Organization
ICU	intensive care unit
MuLBSTA	multilobar infiltrates, lymphopenia, bacterial co-infection, smoking history, hypertension, and age ≥ 65 years
FiO_2_	fraction of inspired oxygen
HFNO	high-flow nasal oxygen
NIMV	non-invasive mechanical ventilation
IMV	invasive mechanical ventilation
LDH	lactate dehydrogenase
IL-6	interleukin-6
SD	standard deviation
IQR	interquartile range
OR	odds ratio
CI	confidence interval
COPD	chronic obstructive pulmonary disease
