# Supplementary material for: Lung Ultrasound Abnormalities and LUS Score After COVID-19 Pneumonia: Determinants and Associations with Dyspnoea in a Prospective Cohort
Source: J Clin Med. 2026 Apr 30;15(9):3438. doi: 10.3390/jcm15093438 (PMC13164168; doi:10.3390/jcm15093438)
Supplement: Supplementary file 1 [file jcm-15-03438-s001.zip › jcm-4258713-supplementary.pdf]

Supplementary materials

Supplementary Table S1

Table S1. Clinical, laboratory, acute-phase, and symptom-related factors associated with persistent lung ultrasound abnormalities (univariable analysis)

Variable		Pleural line irregularity			⩾ 3 B-lines per region			Subpleural consolidations		
		**Present**	**Absent**	***p***	**Present**	**Absent**	***p***	**Present**	**Absent**	***p***
**Baseline characteristics**										
Age, years		66.6 ± 13.1	59.4 ± 15.3	**< 0.001**	69.5 ± 11.2	62.0 ± 14.8	**< 0.001**	67.1 ± 13.1	63.7 ± 14.3	**< 0.05**
Male sex, n (%)		120 (83.9)	23 (16.1)	**0.001**	73 (51.0)	70 (49.0)	**< 0.001**	58 (40.6)	85 (59.4)	0.089
Female sex, n (%)		78 (66.1)	40 (33.9)	**0.001**	27 (22.9)	91 (77.1)	**< 0.001**	35 (29.7)	83 (70.3)	0.089
Body mass index, kg/m^2		28.1 ± 4.8	30.3 ± 6.7	**0.002**	28.1 ± 4.3	28.9 ± 5.9	0.120	27.7 ± 4.4	29.1 ± 5.8	**0.016**
Smoking history, n (%)		9 (47.4)	10 (52.6)	**0.006**	6 (31.6)	13 (68.4)	0.702	4 (21.1)	15 (78.9)	0.259
Arterial hypertension, n (%)		124 (82.1)	27 (17.9)	**0.009**	69 (45.7)	82 (54.3)	**0.006**	59 (39.1)	92 (60.9)	0.192
Diabetes mellitus, n (%)		62 (74.7)	21 (25.3)	0.758	36 (43.4)	47 (56.6)	0.275	35 (42.2)	48 (57.8)	0.165
Dyslipidaemia, n (%)		82 (78.8)	22 (21.2)	0.379	45 (43.3)	59 (56.7)	0.195	40 (38.5)	64 (61.5)	0.509
Hypertensive heart disease, n (%)		22 (100)	0 (0)	**0.012**	10 (45.5)	12 (54.5)	0.498	9 (40.9)	13 (59.1)	0.644
Ischaemic heart disease, n (%)		26 (89.7)	3 (10.3%)	0.069	18 (62.1)	11 (37.9)	**0.010**	12 (41.4)	17 (58.6)	0.540
Chronic obstructive pulmonary disease, n (%)		12 (100)	0 (0)	0.076	11 (91.7)	1 (8.3)	**< 0.001**	8 (66.7)	4 (33.3)	**0.047**
Active malignancy, n (%)		8 (57.1)	6 (42.9)	0.110	3 (21.4)	11 (78.6)	0.260	6 (42.9)	8 (57.1)	0.576
Immunosuppressive condition, n (%)		12 (75.0)	4 (25.0)	1.000	6 (37.5)	10 (62.5)	1.000	8 (50.0)	8 (50.0)	1.000
**Acute COVID-19 pneumonia characteristics**										
Severity of pneumonia n (%)	Very mild–mild	91 (61.5)	57 (38.5)	**< 0.001**	24 (16.2)	124 (83.8)	**< 0.001**	37 (25.0)	111 (75.0)	**< 0.001**
	Moderate–very severe	107 (94.7)	6 (5.3)		76 (67.3)	37 (32.7)		56 (49.6)	57 (50.4)	
MuLBSTA score		10.2 ± 3.8	7.1 ± 3.6	**< 0.001**	11.8 ± 3.2	7.9 ± 3.7	**< 0.001**	10.6 ± 3.8	8.8 ± 3.9	**< 0.001**
MuLBSTA ≥ 12		76 (93.8)	5 (6.2)	**< 0.001**	58 (71.6)	23 (28.4)	**< 0.001**	42 (51.9)	39 (48.1)	**< 0.001**
**Laboratory parameters during acute phase**										
Lymphocytes, cells/mm^3		770 (590–1135)	990 (670–1360)	**< 0.001**	695 (532.5–920)	960 (650–1310)	**< 0.001**	750 (590–1120)	850 (600–1270)	0.129

D-dimer, ng/mL	1256 (630–3759)	655 (429–1434)	**< 0.001**	1810 (822–4115)	882 (502–1777)	**< 0.001**	1811 (889–4967)	880 (506–1941)	**< 0.001**
LDH, U/L	316 (247–411)	268 (202–340)	**< 0.001**	337 (262–430)	281 (225–350)	**< 0.001**	319.5 (249–402)	283 (236–371)	0.080
Ferritin, ng/mL	683.4 (342–1373)	543 (158–952)	**< 0.05**	712 (421–1419)	573 (217–1187)	**< 0.01**	887.2 (368.8–1472)	583.1 (259–1083)	**< 0.02**
**Clinical features at follow-up**									
Dyspnoea (mMRC) mMRC = 0, n (%)	57 (66.3)	29 (33.7)	**0.011**	22 (25.7)	64 (74.4)	**0.003**	20 (23.3)	66 (76.7)	**0.005**
mMRC ⩾1, n (%)	141 (80.6)	34 (19.4)		78 (44.6)	97 (55.4)		73 (41.7)	102 (58.3)	
Fatigue, n (%)	136 (80.5)	33 (19.5)	**0.023**	68 (40.2)	101 (59.8)	0.425	67 (39.6)	102 (60.4)	0.079
Chest pain, n (%)	44 (86.3)	7 (13.7)	0.067	17 (33.3)	34 (66.7)	0.521	23 (45.1)	28 (54.9)	0.142
Cough, n (%)	35 (85.4)	6 (14.6)	0.163	20 (48.8)	21 (51.2)	0.162	15 (36.6)	26 (63.4)	1.000
Weight loss, n (%)	33 (89.2)	4 (10.4)	**0.040**	19 (51.4)	18 (48.6)	0.100	24 (64.9)	13 (35.1)	**< 0.001**

Data are presented as mean ± standard deviation, median [interquartile range], or number (%), as appropriate. Comparisons were performed using χ^2 test or Mann–Whitney U test as appropriate. Statistically significant values ($p < 0.05$) are shown in bold.
Immunosuppressive condition includes patients receiving immunosuppressive therapy or with underlying immunosuppressive conditions.
Abbreviations: LDH, lactate dehydrogenase.

Supplementary Table S2

Table S2. Clinical, laboratory, acute-phase, and symptom-related factors associated with elevated LUS score (>6) (univariable analysis).

Variable	LUS score ≤ 6	LUS score > 6	*p*
**Baseline characteristics**			
Age, years	60.9 ± 14.8	69.7 ± 11.1	**< 0.001**
Male sex, n (%)	63 (44.1)	80 (55.9)	**< 0.001**
Female sex, n (%)	80 (67.8)	38 (32.2)	**< 0.001**
Body mass index, kg/m2	29.3 ± 6.2	27.8 ± 4.1	**0.011**
Smoking history, n (%)	13 (68.4)	6 (31.6)	0.317
Former smokers, n (%)	37 (43.0)	49 (57.0)	**0.011**
Arterial hypertension, n (%)	74 (49.0)	77 (51.0)	**0.038**
Diabetes mellitus, n (%)	39 (47.0)	44 (53.0)	0.111
Dyslipidaemia, n (%)	51 (49.0)	53 (51.0)	0.164
Hypertensive heart disease, n (%)	9 (40.9)	13 (59.1)	0.253
Ischaemic heart disease, n (%)	10 (34.5)	19 (65.5)	**< 0.05**
Chronic obstructive pulmonary disease, n (%)	2 (16.7)	10 (83.3)	**< 0.05**
Active malignancy, n (%)	5 (35.7)	9 (64.3)	0.647
Immunosuppressive condition, n (%)	9 (56.3)	7 (43.8)	0.511

**Acute COVID-19 pneumonia characteristics**				
Severity of pneumonia	Very mild–mild	112 (75.2)	36 (24.3)	**< 0.001**
	Moderate–very severe	31 (27.4)	82 (72.6)	
MuLBSTA score, mean ± SD		7.67 ± 3.7	11.58 ± 3.2	**< 0.001**
MuLBSTA score ≥ 12, n (%)		53 (29.4)	65 (80.2)	**< 0.001**
Requirement for oxygen therapy, n (%)		98 (46.7)	112 (53.3)	**< 0.001**
Duration of oxygen therapy, days, median (IQR)		5 (3–8.2)	8.5 (5–16)	**< 0.001**
Length of hospital stay, days, median (IQR)		7 (5–11)	10.5 (6.75–16)	**< 0.001**
**Laboratory parameters, median (IQR)**				
Lymphocytes (cells/mm³)		980 (670–1325)	690 (545–920)	**< 0.001**
D-dimer (ng/mL)		790 (451–1676)	1830 (868–4202)	**< 0.001**
LDH (U/L)		271 (219–350)	334.5 (266–429)	**< 0.001**
Ferritin (ng/mL)		560.2 (212.5–1061.1)	734 (378.6–1505.5)	**< 0.01**
Interleukin-6 (pg/mL)		7.6 (3.4–15.7)	11.5 (5.9–27.6)	**< 0.02**
**Clinical features at follow-up**				
Dyspnoea (mMRC)	mMRC =0, n (%)	67 (77.9)	19 (22.1)	**< 0.001**
	mMRC ⩾1, n (%)	76 (43.4)	99 (56.6)	
Increase in dyspnoea (Δ mMRC[1] ⩾ 1)	No, n (%)	95 (66.9)	47 (33.1)	**< 0.001**
	Yes, n (%)	48 (40.3)	71 (59.7)	
Fatigue, n (%)		83 (49.1)	86 (50.9)	**0.018**
Chest pain, n (%)		28 (54.9)	23 (45.1)	1.000
Cough, n (%)		15 (36.6)	26 (63.4)	**0.017**
Weight loss, n (%)		13 (35.1)	24 (64.9)	**0.016**

Data are presented as mean ± standard deviation (SD), median (IQR), or number (percentage), as appropriate. Comparisons were performed using the χ^2 test or Fisher's exact test for categorical variables and Student's t-test or Mann–Whitney U test for contin-uous variables, as appropriate. Statistically significant values ($p < 0.05$) are shown in bold.

Immunosuppressive condition includes patients receiving immunosuppressive therapy or with underlying immunosuppressive conditions.

[1]ΔmMRC ⩾ 1 indicates worsening dyspnoea from baseline.

Abbreviations: SD, standard deviation; IQR, interquartile range; LDH, lactate dehydrogenase.

Supplementary Figure S1

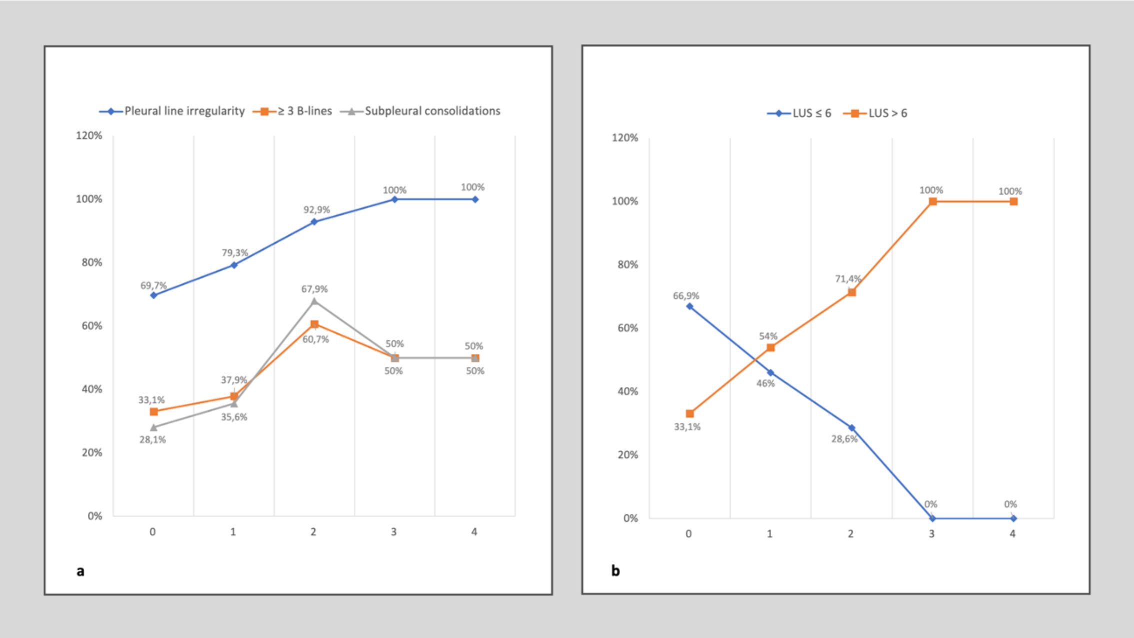

Figure S1. Relationship between worsening dyspnoea and residual lung ultrasound findings at follow-up. (**a**) Frequency of qualitative lung ultrasound abnormalities according to the increase in dyspnoea severity from baseline to follow-up (ΔmMRC 0–4). (**b**) Distribution of LUS score ≤6 and LUS score >6 according to the increase in dyspnoea se-verity (ΔmMRC 0–4). A progressive increase in the frequency of lung ultrasound abnormalities is observed with increasing dyspnoea severity, suggesting a graded association between dyspnoea severity and ultrasound abnormalities.
